# A curcumin analogue GO‐Y030 depletes cancer stem cells by inhibiting the interaction between the HSP70/HSP40 complex and its substrates

**DOI:** 10.1002/2211-5463.13550

**Published:** 2023-01-24

**Authors:** Maya Suzuki, Yohei Yamamoto, Aki Nishijima‐Matsunobu, Yohei Kawasaki, Hiroyuki Shibata, Yasufumi Omori

**Affiliations:** ^1^ Department of Molecular and Tumour Pathology Akita University Graduate School of Medicine Japan; ^2^ Department of Otorhinolaryngology and Head‐and‐Neck Surgery Akita University Graduate School of Medicine Japan; ^3^ Department of Clinical Oncology Akita University Graduate School of Medicine Japan

**Keywords:** cancer stem cells, curcumin analogue, heat shock proteins, HSP70 inhibitors, protein refolding, stress response

## Abstract

Cancer stem cells (CSCs) are proposed to be involved in tumor initiation and play important roles in cancer relapse, metastasis, and drug resistance. Therefore, the targeting of CSCs has potential for effective anticancer therapies. Curcumin is one of the most widely characterized phytochemicals with tumor‐suppressive potential. GO‐Y030 is a novel curcumin analogue exhibiting a much stronger growth‐inhibitory effect than curcumin. In the present study, we verified the potency of GO‐Y030 against a CSC population. We observed that GO‐Y030 suppressed CSC sphere‐forming ability in several cancer cell lines. Interestingly, a specific inhibitor of heat shock protein (HSP) 70 also exhibited effects similar to GO‐Y030 (i.e. inhibition of CSC sphere formation and upregulation of HSP70 and HSP40 protein expression), suggesting that HSP70 and/or HSP40 might be target molecules of GO‐Y030. We then performed an *in vitro* HSP70/HSP40‐mediated refolding activity assay and observed that chaperone activity was efficiently inhibited by GO‐Y030. Finally, we performed a substrate‐binding assay to show that GO‐Y030 reduced the binding of both HSP70 and HSP40 with their substrates. HSPs prevent denaturation or unfolding of client proteins under stressful conditions such as high temperature. Because CSCs by nature adapt to various stresses by reinforcing protein‐folding activity, the function of HSP70/HSP40 is important for the maintenance of CSC population. Our data suggest that GO‐Y030 may impair stress tolerance in CSCs by inhibiting the interaction of HSP70/HSP40 with their substrate proteins and disrupting the function of HSP70/HSP40, thereby contributing to a reduction of the CSC population.

## Graphical abstract

Cancer stem cells (CSCs) are, in general, under cellular stress because of the accumulation of unfolded and/or misfolded proteins, which are usually refolded by heat shock proteins (HSPs) such as HSP70‐1A, resulting in efficient survival of CSCs as an adaptive stress response. GO‐Y030, a curcumin analogue, directly impairs the unfolded protein response exerted by HSPs and causes failure of CSC self‐renewal.

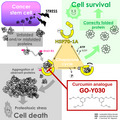

**Abbreviations**
CHX: cycloheximide
CMLA: carboxymethylated α‐lactalbumin
CSC: cancer stem cell
GSEA: Gene Set Enrichment Analysis
HSP: heat shock protein
PBS: phosphate‐buffered saline
PFT‐μ: pifithrin‐μ

A large body of literature has supported the existence of cancer stem cells (CSCs) as a subpopulation of cancer cells [1, 2, 3]. In this model, the cancer tissue consists of a heterogeneous cell population organized in a hierarchical manner sustained by CSCs at the apex. CSCs have the potential to self‐renew as well as to produce non‐CSCs and are considered to be tumor‐initiating cells. Furthermore, CSCs are resistant to chemoradiotherapy because of their infrequent replication, robust DNA repairing, active drug efflux system, and increased defenses against reactive oxygen species. Therefore, strategies targeting CSCs are crucial for effective anticancer therapies.

Curcumin (diferuloylmethane) isolated from the rhizome of the plant turmeric (*Curcuma longa*) is one of the most widely characterized phytochemicals (Fig. 1A). It is well known to regulate multiple transcription factors, including nuclear factor‐κB [4], activator protein‐1 [5], β‐catenin [6, 7], and signal transducer and activator of transcription 3 [8, 9], resulting in inhibition of cancer cell proliferation, invasion, metastasis, and angiogenesis, as well as eradication of CSC population [10, 11, 12]. However, its bioavailability is limited as a result of poor absorption [13]. To newly create possible candidates for a more potent analogue of curcumin and to overcome this disadvantage, Ohori *et al*. [14] screened their synthetic organic compound library. Among approximately 80 new derivatives, GO‐Y030, (1*E*,4*E*)‐1,5‐*bis*[3,5‐*bis*(methoxymethoxy)phenyl]penta‐1,4‐dien‐3‐one (Fig. 1A), is one of the most promising curcumin analogues having potent antitumor activities. Consistent with a prvious study [15], we show here that GO‐Y030 can effectively deplete the CSC population in several cancer cell lines.

**Fig. 1:**
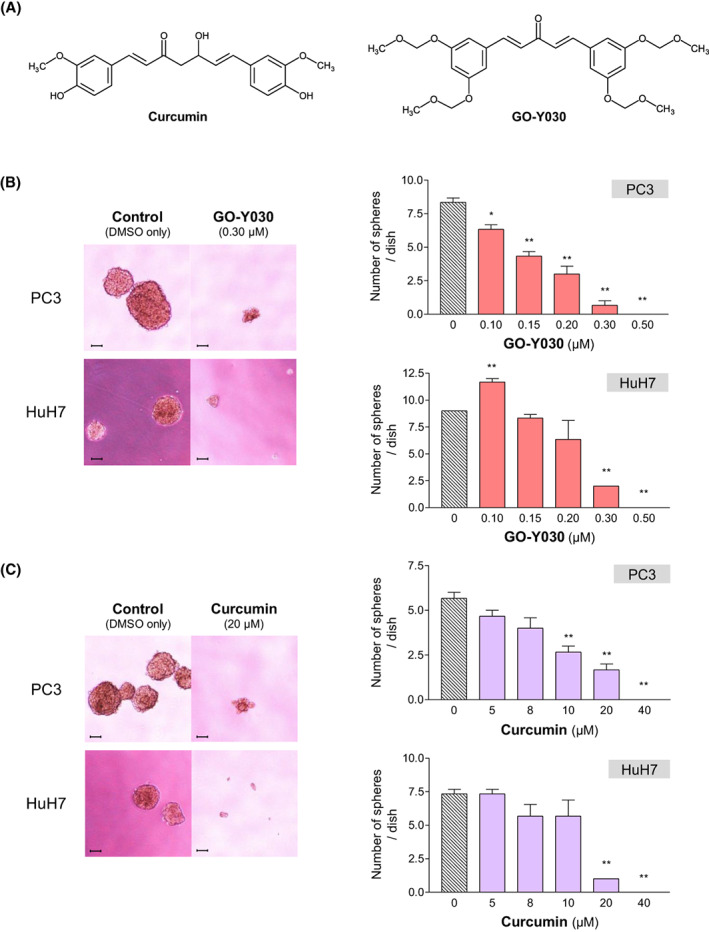
(A) Chemical structures of curcumin and its analog GO‐Y030. (B, C) Effects of GO‐Y030 on CSC sphere‐forming ability of the cancer cell lines. PC3 and HuH7 cells were plated as single cells on ultra‐low attachment 24‐well plates at a density of 200 cells per well in serum‐free medium at 37 °C for 7 and 12 days, respectively. Statistical significance was calculated using Student's *t*‐test. Data represent the mean ± SEM of three independent experiments. *P < 0.05 and **P < 0.01 compared to the control group (dimethylsulfoxide alone). Scale bar = 100 μm.

In general, CSCs are under unfavorable conditions including hypoxia, nutrient deprivation, oxidative stress, and endoplasmic reticulum stress, and thus need to have more efficient mechanisms to tolerate these stresses compared to differentiated non‐CSCs. The stress response is often characterized by the expression of heat shock proteins (HSPs), which are a highly conserved family of molecular chaperones and facilitate the proper folding of nascent proteins and the refolding of denatured proteins. It is thus predicted that HSPs should have a profound influence on CSCs. Several studies have indeed demonstrated that the expression of HSPs is significantly increased in isolated murine and human CSCs [16, 17, 18, 19] and that pharmacological and genetic inhibition of HSPs targets CSCs [20, 21, 22]. Moreover, in the present study, we have found out that GO‐Y030 behaves similarly to the specific inhibitor of HSP70 in terms of CSC sphere formation and HSP70/HSP40‐mediated substrate refolding activity, such that both compounds inhibited these two cellular functions. Our overall data eventually indicate that GO‐Y030 efficiently inhibits the substrate‐binding activity of the HSP70/HSP40 complex, resulting in suppression of CSC sphere formation. To our knowledge, this is the first study elucidating a direct molecular reaction of GO‐Y030 in terms of its anticancer effect.

## Materials and methods

### Cell line and reagents

Human prostatic carcinoma cell line PC3 was obtained from the American Type Culture Collection (Manassas, VA, USA). Human hepatocellular carcinoma cell line HuH7 and human uterine cervical carcinoma cell line HeLa were supplied by Cell Resource Center for Biomedical Research, Institute of Development, Aging and Cancer, Tohoku University (Sendai, Japan). The 293T cell line, which is a highly transfectable derivative of human embryonic kidney cell line 293, was obtained from Clontech Laboratories, Inc. (Takara Bio USA, Inc., Mountain View, CA, USA). PC3, HuH7 and HeLa cells were maintained in RPMI‐1640 medium (Nissui Pharmaceutical, Tokyo, Japan) supplemented with 10% fetal bovine serum (Gibco, Thermo Fisher Scientific, Waltham, MA, USA), 2 mm l‐glutamine, 2.4 g·L^−1^ NaHCO_3_, 100 units·mL^−1^ penicillin, and 100 μg·mL^−1^ streptomycin. The 293T cells were maintained in Dulbecco's modified Eagle's medium (Gibco), 10% fetal bovine serum, 100 units·mL^−1^ penicillin, and 100 μg·mL^−1^ streptomycin. Cells were incubated at 37 °C under a humidified atmosphere of 5% CO_2_. Pifithrin‐μ (PFT‐μ), VER‐155008, and JG98 were purchased from Tocris Bioscience (Bio‐Techne Corp., Minneapolis, MN, USA), Cayman Chemical Company (Ann Arbor, MI, USA), and Selleck Chemicals (Houston, TX, USA), respectively. These compounds were dissolved in dimethylsulfoxide at 10–100 mm as a stock solution.

### CSC sphere‐forming assay

Cancer stem cells sphere‐forming assay was carried out as described previously [23]. Briefly, PC3 and HuH7 cells were plated as single cells on ultra‐low attachment 24‐well plates (Corning, Corning, NY, USA) at a density of 200 cells per well in 1.5 mL of Dulbecco's modified Eagle's medium supplemented with 1% B27 and N2 supplement (Invitrogen, Thermo Fisher Scientific), subsequently incubated in a 5% CO_2_ incubator at 37 °C for 7 days and 12 days, respectively. The number of spheres was counted using a phase contrast microscope. In this study, only colonies with a diameter of 100 μm or greater were counted as spheres.

### Immunoblotting analysis

Immunoblotting analysis was performed mostly as described previously [24]. Cells were grown in 60 mm dishes and lysed in CelLytic M Cell Lysis Reagent (Sigma‐Aldrich, MERCK KGaA, Darmstadt, Germany). Some 8 μg of cell extracts was separated by SDS/PAGE and then transferred to 0.45‐μm polyvinylidene difluoride membranes (Amersham, Cytiva, Eysins, Switzerland) at 1.9 mA·cm^−2^ for 1.5 h with a semidry transfer cell (ATTO, Tokyo, Japan). After blocking with 5% non‐fat skim milk in Tris‐buffered saline with 0.05% Tween‐20, a membrane was incubated with a primary antibody of interest for 1.5 h at room temperature, then with horseradish peroxidase‐conjugated secondary antibody for 1 h at room temperature. The membrane was washed in Tris‐buffered saline with 0.05% Tween‐20, and the proteins‐antibody complex was detected using the Amersham ECL detection system (Cytiva). To measure the stability of HSP70‐1A, a cycloheximide (CHX) chase assay was performed. CHX was purchased from Sigma‐Aldrich. PC3 and HeLa cells were treated with 5 μg·mL^−1^ CHX for 6, 9, and 12 h. The whole‐cell lysates were then applied to immunoblotting analysis as described above to quantify HSP70‐1A protein. The primary antibodies used for the immunoblotting analysis were: anti‐HSP70 mAb clone 4E7E5 (Proteintech, Rosemont, IL, USA) (dilution 1 : 10 000); anti‐HSP70 polyclonal antibody (pAb) (Proteintech) (dilution 1 : 4000); anti‐HSC70 mAb clone 1A4B3 (Proteintech) (dilution 1 : 10 000); anti‐HSP90 mAb clone 3F11C1 (Proteintech) (dilution 1 : 8000); anti‐DNAJA1 pAb (Proteintech) (dilution 1 : 800); anti‐DNAJB1 pAb (Proteintech) (dilution 1 : 4000); and anti‐GAPDH mAb clone 6C5 (HyTest Ltd, Turku, Finland) (dilution 1 : 12 000).

### Puromycin incorporation assay

To measure a global protein synthesis, puromycin incorporation assay was performed, where puromycin acts as a tyrosyl‐tRNA mimic that blocks translation by labeling and releasing elongating polypeptide chains from translating ribosomes. The amount of incorporated puromycin thus reflects the extent of polypeptide elongation after initiation of translation. PC3 cells were cultured in presence or absence of 2 μm GO‐Y030 for 24 h. After exposure to 2 μg·mL^−1^ puromycin (Sigma‐Aldrich) for 30 min, puromycin incorporation was detected by immunoblotting analysis using anti‐puromycin mAb clone 12D10 (Sigma‐Aldrich) (dilution 1 : 10 000). Treatment with CHX (50 μg·mL^−1^) was included as a negative control.

### Co‐immunoprecipitation

Co‐immunoprecipitation analysis was performed according to the previously published procedure with some modifications [25]. Total cellular protein (300 μg) was added to 1 μg of anti‐HSP70/HSP72 mAb clone C92F3A‐5 (Enzo Life Sciences, Inc., Farmingdale, NY, USA) or its isotype control, mouse IgG subclass 1 (Medical & Biological Laboratories Co., Ltd, Nagoya, Japan), and incubated at 4 °C for 1 h. Then, 20 μL of Protein A/G PLUS‐Agarose (Santa Cruz Biotechnology, Inc., Dallas, TX, USA) was added and incubated at 4 °C overnight. Agarose beads were washed five times with washing buffer. Immunoprecipitated proteins were analyzed by immunoblotting analysis.

### Luciferase refolding assay in living cells

To test the effect of GO‐Y030 on the refolding activity of intracellular chaperones, the luciferase refolding assay in living cells was designed according to the protocol described by Lazarev *et al*. [26] with some modifications. Briefly, PC3 and 293T cells were transiently transfected with a pGL3‐Control Vector (Promega Corp., Madison, WI, USA) coding luciferase protein using FuGene® HD Transfection Reagent (Promega Corp.) and *Trans*IT®‐293 Transfection Reagent (Mirus Bio LLC, Madison, WI, USA), respectively, at a ratio of 3 : 1 (3 μL of reagent per 1 μg plasmid DNA) in basal medium containing low levels of antibiotics for 24 h. Transfected cells were then treated with GO‐Y030, PFT‐μ or dimethylsulfoxide alone for control and subjected to heat shock at 43 °C for 30 min to denature luciferase. After recovery at 37 °C for 6 h, cells were lysed with Luciferase Cell Culture Lysis Reagent (Promega Corp.). Cell lysate from each sample (0.1 μg) was mixed with 50 μL of luciferin‐containing Luciferase Assay Reagent (Promega Corp.) and luciferase activity was measured using a TECAN Infinite 200 microplate reader (TECAN Group Ltd, Männedorf, Switzerland). Average luminescence was measured as counts per second from three independent experiments and the data were presented as a percentage of the refolding activity. The refolding activity of the control treated with dimethylsulfoxide alone was set as 100%.

### Cell‐free assay of HSP70/HSP40‐mediated refolding activity

To investigate the effect of GO‐Y030 on HSP70/HSP40‐mediated chaperone activity in the cell‐free system, we employed an HSP70/HSP40 Glow‐Fold Protein Refolding Kit (Boston Biochem, Bio‐Techne Corp.) and performed an assay in accordance with the manufacturer's protocol with modifications. In brief, Glow‐Fold Substrate protein equivalent to luciferase protein was mixed with HSP70/HSP40 complex, ATP, and the compounds to be examined or dimethylsulfoxide alone. The concentration of the HSP70 inhibitors was as: PFT‐μ, 0.8 mm; VER‐155008, 0.4 mm; and JG98, 5 μm. The reactions were exposed to 45 °C for 7 min and then placed in 30 °C to initiate refolding reactions. After the indicated times, aliquots taken from each of the reactions were mixed with luciferin reagent and luciferase activity was measured using a TECAN Infinite 200 microplate reader. Average luminescence was measured as counts per second from three independent experiments and the data are presented as a percentage of the refolding activity. The refolding activity of each sample before heat shock was set as 100%.

### Substrate‐binding activity assay

The chaperone activity of HSP70 was measured in accordance with the protocol described by Lazarev *et al*. [26, 27] with some modifications. Carboxymethylated lactalbumin (CMLA) as a substrate target protein was prepared as described previously [28, 29]. For this, bovine α‐lactalbumin (Sigma‐Aldrich, MERCK KGaA) was dissolved at 1 mg·mL^−1^ in 1 m Tris–HCl pH 8.0 containing 8 m urea and 20 mm dithiothreitol. An alkylation reaction was performed using iodoacetamide at a final concentration of 40 mm and quenched after 30 min by adding 10 mm dithiothreitol at the final concentration. Finally, the solution was dialyzed twice against 1 L of phosphate‐buffered saline (PBS) for 2 h each. The resultant CMLA was immobilized onto the wells of a 96‐well plate at a concentration of 500 ng per well in 50 μL of PBS. The wells were rinsed three times with water and coated with the blocking buffer, PBS containing 0.2% (w/v) BSA, at room temperature for 1 h. Next, 50 ng per well of recombinant human HSP70‐1A protein (StressMarq Biosciences Inc., Victoria, BC, Canada) or recombinant human HSP40/Hdj1 (dnaJ‐B1) protein (Enzo Life Sciences Inc.) in the reaction buffer (20 mm Tris–HCl (pH 7.5), 20 mm NaCl, and 10 mm MgCl_2_) supplemented with 0.2% BSA was added to each well. After 60 min of incubation at 30 °C, the wells were rinsed with water and incubated with 50 μL of anti‐HSP70 pAb (Proteintech) (dilution 1 : 5000) or anti‐DNAJB1 pAb (Proteintech) (dilution 1 : 5000) in the blocking buffer at room temperature for 2 h. After three washes, the wells were probed at room temperature for 2 h with alkaline phosphatase‐labeled goat anti‐rabbit IgG (SeraCare Life Sciences, Inc., Milford, MA, USA) (dilution 1 : 2500) in the blocking buffer. After another three washes, BluePhos Microwell substrate solution (SeraCare Life Sciences, Inc.) was added to each well. Absorbance was measured at 620 nm using a TECAN Sunrise absorbance microplate reader (TECAN Group Ltd).

### Oligo DNA microchip array analysis for mRNA expression and gene set enrichment analysis (GSEA)

A total RNA was isolated from PC3 cells after 24‐h incubation with 0.8 μm GO‐Y030 or dimethylsulfoxide alone using TRIzol™ Reagent (Invitrogen, Thermo Fisher Scientific) in accordance with the manufacturer's instructions. Microarray analyses were performed using the 3D‐Gene® Human Oligo chip 25 k (Toray Industries, Inc., Tokyo, Japan). GSEA was performed using these microarray data with the gsea, version 4.3.2 (Broad Institute of Massachusetts Institute of Technology and Harvard; https://www.gsea‐msigdb.org/gsea/index.jsp) [1]. We examined enrichment of four gene sets with different annotations including ‘translation’ (GO:0006412), ‘positive regulation of translation’ (GO:0045727), ‘mRNA transcription’ (GO:0009299), and ‘transcription initiation at RNA polymerase II promoter’ (GO:0006367). *P* < 0.05 was considered statistically significant for normalized enrichment scores.

### Antibody‐sandwich ELISA

An antibody‐sandwich ELISA was used for detection of extracellular HSP70‐1A in culture medium. Briefly, PC3 cells were incubated at 37 °C for 24 h in RPMI 1640 medium with or without 2 μm GO‐Y030. The conditioned medium was collected and centrifuged at 450 *g* at 4 °C for 10 min and the supernatant was stored. 96‐well microtiter ELISA plates (Sumitomo Bakelite Co., Ltd, Tokyo, Japan) were coated with anti‐HSP70 mouse mAb clone 4E7E5 (Proteintech, 2 μg·mL^−1^) in 0.1 m carbonate–bicarbonate buffer (pH 9.6) at 4 °C overnight. The wells were rinsed three times with water and coated with the blocking buffer, PBS containing 0.2% (w/v) BSA, at room temperature for 30 min. After three washes, samples were added to the wells and incubated at room temperature for 2 h. The wells were rinsed with water and incubated with 50 μL of anti‐HSP70 rabbit pAb (Proteintech) (dilution 1 ∶ 800) at room temperature for 2 h. The detection procedure is the same as the substrate‐binding activity assay.

### Statistical analysis

Data represent the mean ± SEM of at least three independent experiments. Significant differences were analyzed using Student's *t*‐test to compare two groups. *P* < 0.05 was considered statistically significant.

## Results

### GO‐Y030 can efficiently suppress the sphere‐forming ability of the cancer cell lines

To verify the potency of GO‐Y030 against the CSC population, we performed a CSC sphere formation assay using the human prostatic carcinoma cell line PC3 and the human hepatocellular carcinoma cell line HuH7. In both cell lines, GO‐Y030 reduced the number of spheres in a dose‐dependent manner (Fig. 1B). Although curcumin also suppressed sphere‐forming ability, it required an approximately 50‐fold higher dose to attain the same effect as GO‐Y030 (Fig. 1C). Consistently with previous studies [15], these results indicate that GO‐Y030 can potently reduce the CSC population and is more effective than curcumin.

### GO‐Y030 decreases HSP70/HSP40‐mediated refolding activity in living cells

**Fig. 2:**
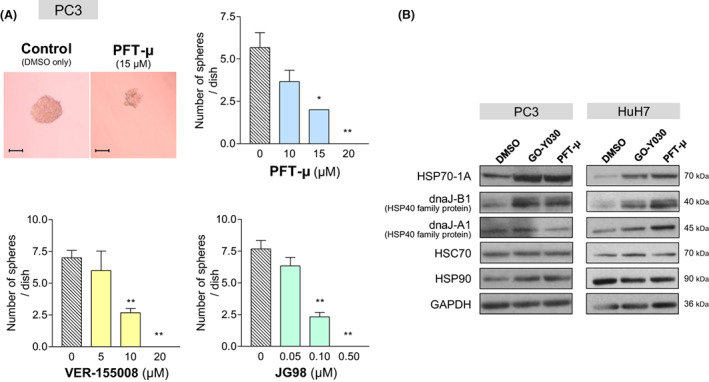
Comparison of GO‐Y030 with inhibitors of HSP70‐1A, in terms of CSC sphere formation and expression of HSP family proteins. (A) Effects of PFT‐μ (upper), VER‐155008 (lower left), and JG98 (lower right) on PC3 CSC sphere forming ability. All of these HSP70 inhibitors significantly reduced the number of spheres. Statistical significance was calculated using Student's *t*‐test. Data represent the mean ± SEM of three independent experiments. *P < 0.05 and **P < 0.01 compared to the control group (dimethylsulfoxide alone). Scale bar = 100 μm. (B) PC3 and HuH7 cells were treated with GO‐Y030 (2 μm) or PFT‐μ (20 μm). Twenty‐four hours later, cell lysates were applied for immunoblotting analysis using antibodies to the indicated HSP family proteins. GAPDH was used as a loading control. Although both compounds increased expression of HSP70‐1A and dnaJ‐B1, the other HSP families did not exhibit any changes.

In CSCs of various human cancers, several HSP family members including HSP70‐1A [19], dnaJ‐B8 [21], HSP90 [30], glucose‐regulated protein of 78‐kDa (GRP78) [31], and HSP27 [32], are overexpressed and considered to play a pivotal role in CSC development and maintenance. Moreover, there have been several studies describing the CSC‐targeting effect of different HSP90 inhibitors [33, 34, 35]. We next examined whether pharmacological inhibition of HSP70 could target CSCs. As shown in Fig. 2A, PFT‐μ (2‐phenylethynesulfonamide) [36], VER‐155008 [37, 38], and JG98 [39], all of which are already known to be small molecule inhibitors of HSP70 family, could inhibit CSC sphere formation.

Accordingly, we speculated that GO‐Y030 could also inhibit the expression and/or function of HSPs, resulting in reduction of CSC populations. To explore the functional relationship of GO‐Y030 to HSPs, we first examined the effects of GO‐Y030 on the expression of HSP family proteins. Although the oligo DNA microchip array analysis for mRNA expression in PC3 cells revealed that all the HSPs examined were significantly decreased in mRNA expression after treatment with GO‐Y030 (Table S1), the protein expression of HSP70‐1A, a principal HSP70 encoded by the gene *HSPA1A*, and dnaJ‐B1, a principal HSP40 encoded by the gene *DNAJB1*, was drastically enhanced, whereas no other HSPs showed any changes (Fig. 2B). Interestingly, the HSP70 inhibitor PFT‐μ exhibited effects similar to those for GO‐Y030 (Fig. 2B), raising the possibility that GO‐Y030 might regulate the function of HSP70‐1A and its co‐chaperones. To explore how both GO‐Y030 and PFT‐μ regulate the expression of *HSPA1A* mRNA and its protein in an inverse manner, the half‐life of HSP70‐1A protein was measured using a CHX‐chase assay. Neither GO‐Y030, nor PFT‐μ could affect half‐life of HSP70‐1A in PC3, HuH7, or HeLa cells (data not shown). Furthermore, it has been reported that HSP70‐1A is released to the outside of the cells as an extracellular HSP70 [40, 41, 42]. To verify the possibility that GO‐Y030 and PFT‐μ inhibit production of the extracellular HSP70, resulting in increase of HSP70‐1A protein inside the cells, HSP70‐1A protein in culture medium was measured with an antibody‐sandwich ELISA method, which revealed that the amount of extracellular HSP70‐1A protein in culture medium was not changed by GO‐Y030 or PFT‐μ (Fig. S1). Taken together, it is concluded that GO‐Y030 and PFT‐μ suppress the transcription of *HSPA1A* mRNA and, at the same time, enhance synthesis of HSP70‐1A protein. In addition, because mRNA expression of the gene sets involved in mRNA transcription and/or protein synthesis, as revealed by GSEA (Fig. S2A), did not significantly change with respect to the presence and absence of GO‐Y030 and because global protein synthesis, as revealed by the puromycin incorporation assay (Fig. S2B), was not affected by the treatment with GO‐Y030, we considered that the increased expression of HSP70‐1A and dnaJ‐B1 proteins did not result from GO‐Y030‐mediated enhancement of global translation but from GO‐Y030‐mediated preferential upregulation of the synthesis of HSP70‐1A and dnaJ‐B1.

The 70‐kDa HSP family consisting of 13 members is divided into two types: the stress‐inducible type represented by HSP70‐1A and the constitutively expressed one including 70‐kDa heat shock cognate protein (HSC70) and GRP78. In normal cells, HSP70‐1A is expressed only at a low or undetectable level under non‐stress conditions and is rapidly induced to increase expression by environmental stress events such as an elevated temperature, oxidative agents, and heavy metals. However, in most cancer cells, HSP70‐1A is known to be constitutively overexpressed even without any stress exposure, implying that HSP70‐1A may play an essential role in the maintenance and progression of cancers. HSP70s usually need to form a complex directly with HSP40 family members to upregulate the chaperone function and promote refolding of their target substrate proteins [43, 44].

**Fig. 3:**
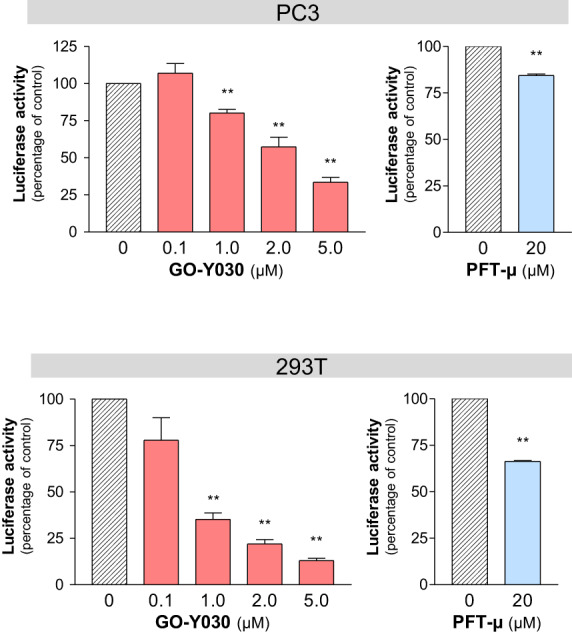
Inhibitory effect of GO‐Y030 on refolding activity of intracellular chaperone. PC3 (upper) and 293T (lower) cells were transiently transfected with an expression construct coding luciferase protein. Transfected cells were treated with GO‐Y030 or PFT‐μ at the indicated concentrations, subjected to heat shock (43 °C, 30 min), and then cultured at 37 °C for 6 h before the luciferase assay. The refolding activity of the control group treated with dimethylsulfoxide alone was set as 100%. Statistical significance calculated using Student's *t*‐test. Data represent the mean ± SEM of three independent experiments. **P < 0.01 compared to the control group (dimethylsulfoxide alone).

Hypothesizing that GO‐Y030 may inhibit protein refolding, we evaluated the refolding efficiency of heat‐denatured luciferase protein exogenously expressed in PC3 and 293T cells in the presence or absence of GO‐Y030. As expected, after luminescence decreased as a result of heat shock, it increased again during the recovery times most probably as a result of HSP‐mediated refolding of luciferase. Figure 3 shows that GO‐Y030 has the ability to disturb the recovery of luciferase activity in a dose‐dependent manner compared to dimethylsulfoxide, suggesting that GO‐Y030 inhibits the chaperoning function of HSPs.

**Fig. 4:**
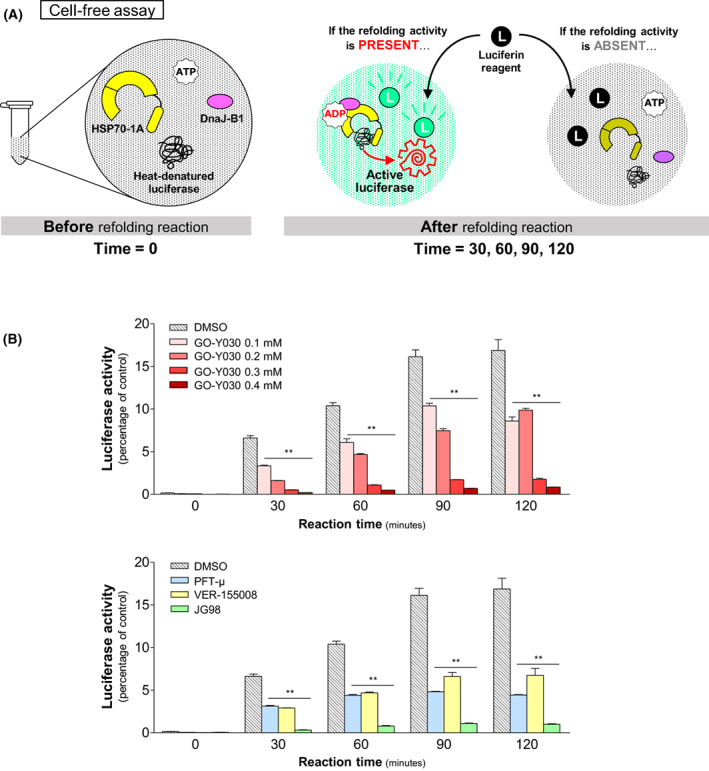
Cell‐free assay of HSP70/HSP40‐mediated refolding activity. (A) Schematic diagram of Substrate‐binding activity assay. (B) Upper: refolding activity was reduced at each time point in the presence of GO‐Y030 in a dose‐dependent manner compared to the the control, whereas misfolded inactive luciferase was efficiently refolded in the absence of GO‐Y030 by recombinant HSP70/HSP40 protein, leading to a rapid recovery of enzymatic activity. The results indicate that GO‐Y030 inhibits the chaperone function of HSP70/HSP40. (B) Lower: all four different HSP70 inhibitors also inhibited the refolding of heat denatured luciferase. The refolding activity of each sample before heat shock was set as 100%. Statistical significance calculated using Student's *t*‐test. Data represent the mean ± SEM of three independent experiments. **P < 0.01 compared to the control group (dimethylsulfoxide alone).

Because this effect of GO‐Y030 was revealed in living cells, there still remains the possibility that GO‐Y030 might inhibit indirectly the function of HSPs by modulating some regulatory factor of HSPs. We thus verified whether GO‐Y030 could inhibit the HSP70/HSP40 protein refolding function in a cell‐free system (Fig. 4A), where the reaction solution contains only six factors, comprising HSP70‐1A, dnaJ‐B1, heat‐denatured luciferase, luciferin, ATP, and one of small compounds including GO‐Y030, PFT‐μ, VER‐155008, and JG98. Figure 4B clearly demonstrates that GO‐Y030 can impair the ability of HSP70/HSP40 complex to refold heat‐denatured luciferase in the cell‐free system in a dose‐dependent manner, indicating that GO‐Y030 has an intrinsic activity to inhibit HSP70/HSP40‐mediated protein refolding and that the HSP70/HSP40 complex is a direct target of GO‐Y030. Furthermore, all three of the inhibitory compounds including PFT‐μ, VER‐155008, and JG98 also exhibited an efficient inhibition of HSP70/HSP40‐mediated protein refolding function (Fig. 4B, lower). Because these compounds have been shown to have an inhibitory effect on CSCs as a result of HSP70 disruption (Fig. 2A), GO‐Y030 probably diminishes the CSC population by impairing the protein refolding activity of HSP70/HSP40.

### GO‐Y030 does not alter the interaction between HSP70 and HSP40

**Fig. 5:**
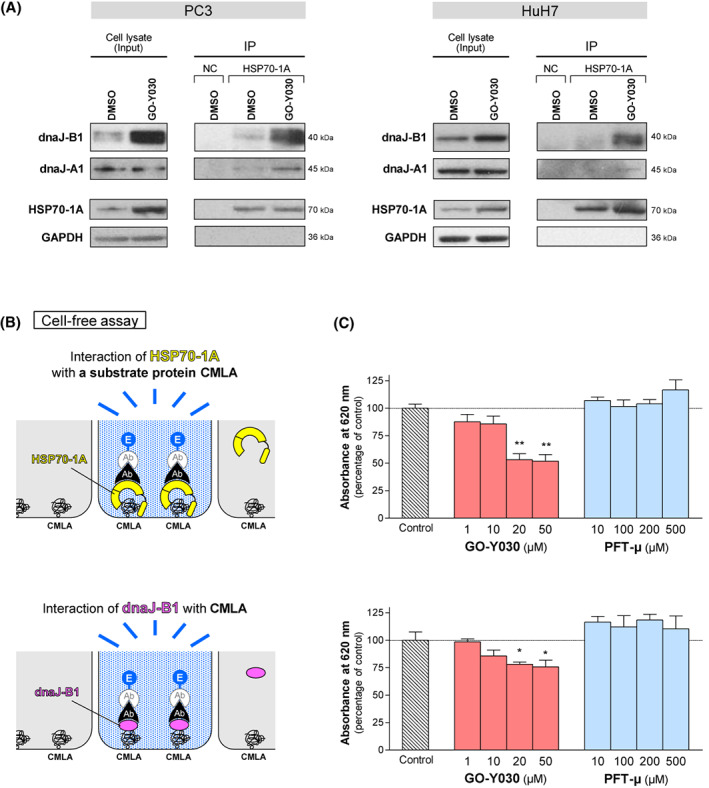
Inhibitory effect of GO‐Y030 on the substrate‐recognition and ‐binding activity of both HSP70‐1A and dnaJ‐B1. (A) Co‐immunoprecipitation (Co‐IP) of HSP70‐1A in PC3 (left) and HuH7 (right) cell lysates. Whole cell lysates were prepared from the cell lines that had been treated with GO‐Y030 (2 μm) or dimethylsulfoxide for 24 h. Immunoprecipitation and western blot analysis using the indicated antibodies revealed that HSP70‐1A interacted with HSP40 family proteins such as dnaJ‐A1 and dnaJ‐B1 even after treatment with GO‐Y030. These data suggest that GO‐Y030 should not affect the association between HSP70‐1A and HSP40s. Input was 3% of total protein extract used in the assay. Negative controls (NC) were performed by using an isotype and concentration matched non‐specific control antibody. (B) Schematic diagram for the substrate‐binding activity assay. Interaction of HSP70‐1A (upper) or dnaJ‐B1 (lower) with the substrate protein carboxymethylated α‐lactalbumin (CMLA), which is permanently unfolded, was measured using a modified enzyme‐linked immunosorbent assay. The substrate‐binding activity was determined as the enzymatic activity of alkaline phosphatase. (C) Although GO‐Y030 reduced the binding of not only HSP70‐1A (upper) but dnaJ‐B1 (lower) to CMLA in a dose‐dependent manner, treatment with PFT‐μ did not show any significant differences. The data are acquired as percentage of absorbance of control group treated with dimethylsulfoxide alone. Statistical significance was calculated using Student's *t*‐test. Data represent the mean ± SEM of three independent experiments. *P < 0.05 and **P < 0.01 compared to the control group (dimethylsulfoxide alone). Ab, antibody; E, enzyme.

Because HSP40 directly binds to HSP70 and modulates the affinity between HSP70 and client proteins [43, 44], we investigated whether GO‐Y030 could block the interaction between HSP70 and HSP40 and inhibit the chaperone activity of the HSP70/HSP40 complex. To explore this, we performed co‐immunoprecipitation experiments. Whole‐cell extracts were prepared from PC3 and HuH7 cells that were treated with or without GO‐Y030 for 24 h. Immunoprecipitation and western blot analysis revealed that, even after treatment with GO‐Y030, HSP40 family proteins dnaJ‐B1 and dnaJ‐A1 were co‐precipitated with HSP70‐1A by the anti‐HSP70 antibody (Fig. 5A). This result indicates that GO‐Y030 does not affect the physical association between HSP70 and HSP40.

### GO‐Y030 reduces the substrate‐binding activity of both HSP70 and HSP40

We next examined whether GO‐Y030 could suppress the capacity of HSPs to recognize and bind to a denatured protein. To test the affinity of HSPs for protein substrate, we employed a substrate‐binding assay based on previous work by Lazarev *et al*. [26, 27] (Fig. 5B). HSP70s generally recognize nascent polypeptide chains, peptides in the extended conformation, and unfolded proteins. In place of denatured substrates, we used CMLA, which mimics a nascent polypeptide substrate and remains permanently unfolded [28, 45, 46]. As a consequence, GO‐Y030 reduced the binding of HSP70‐1A to CMLA in a dose‐dependent manner, whereas treatment with PFT‐μ did not demonstrate any significant differences (Fig. 5C, upper). Besides HSP70‐1A, binding of dnaJ‐B1 to CMLA was also blocked by GO‐Y030 (Fig. 5C, lower). Taken together, our data indicate that GO‐Y030 reduces the substrate‐binding activity of both HSP70 and HSP40, resulting in the inhibition of HSP70/HSP40 chaperone function.

## Discussion

Cancer cells, in general, tend to express a high level of HSP70 protein constitutively, regardless of the extent of environmental and intracellular stresses that often elevate the frequency of protein misfolding. Molecular chaperones such as HSP70 help cancer cells, notably the CSC subpopulation, to adapt to hostile environmental stresses such as hypoxia, nutrient deprivation, and oxidative stress, as well as to overcome these severe conditions by catalyzing the proper folding of nascent proteins and the refolding of denatured proteins, and thus are considered to be central to the adaptive cellular response contributing to maintaining the cell viability. Several review articles have recently described the importance of HSPs for the CSC population [17, 18], with a proteomics analysis revealing that HSPs were significantly upregulated in the mammospheres from breast cancer cell lines [19]. More recently, Hyun *et al*. [47] have demonstrated that enforced expression of HSP70 or HSP90 in non‐small cell lung cancer cells promoted the acquisition of CSC phenotypes and that a natural product named evodiamine, which directly binds to and destabilizes HSP70, displays potent anti‐CSC activities *in vitro* and *in vivo*. Taken together, it appears that cancer cells exploit HSP‐mediated adaptive response not only to maintain, but also to expand the CSC population.

From this point of view, it is quite rational that, as revealed by the present study, GO‐Y030 impairs function of HSPs by preventing them from binding to their substrates, leading to a reduction of the CSC population. It has been reported that different anti‐oncogenic effects of curcumin and its derivatives are mediated by quantitative or functional downregulation of several oncogenic transcription factors including nuclear factor‐κB [4], activator protein‐1 [5], β‐catenin [6, 7], and signal transducer and activator of transcription 3 [8, 9]. However, it is still unclear whether these molecules are direct targets of curcumin and its derivatives. Considering that their molecular structures are quite different from molecule to molecule and that the effects of protein modification such as phosphorylation are also different from molecule to molecule or opposite to each other, we assume that the downregulation of these transcription factors should be one of the downstream effects in signal transduction cascades but not a direct or primary action of curcumin and its derivatives. By contrast, GO‐Y030‐mediated inhibition of HSP70/HSP40 functions including substrate refolding and binding to the substrates were revealed by our cell‐free experimental systems (Figs 4 and 5B,C). For the first time, our present study has identified HSP70‐1A and dnaJ‐B1 as direct target molecules of GO‐Y030 and demonstrated that the inhibition of HSP70/HSP40 function is the primary action of GO‐Y030 for its tumor‐suppressive effects.

The most intriguing finding in the present study is that, although GO‐Y030 decreased *HSPA1A* and *DNAJB1* mRNAs (Table S1), the same reagent increased HSP70‐1A and dnaJ‐B1 proteins with respect to expression (Fig. 2B) but nevertheless inhibited the function of these proteins (Figs 4B and 5C). Similar effects were also realized by the several established HSP70 inhibitors (Figs 2B, 3, 4, and 5C). Because our experiments excluded the possibilities that GO‐Y030 could prolong the half‐life of HSP70‐1A and that GO‐Y030 could inhibit the release of the extracellular HSP70‐1A (Fig. S1), it is most likely that GO‐Y030 enhanced the translation of HSP70‐1A protein. In any case, GO‐Y030 impaired the function of HSP70‐1A and dnaJ‐B1 proteins, as did PFT‐μ. We assume that the GO‐Y030‐mediated increase of HSP70‐1A and dnaJ‐B1 proteins should be irrelevant to anti‐oncogenic action of GO‐Y030.

One of the peculiar features of GO‐Y030 is that it sensitizes tumor cells to various cellular stresses. GO‐Y030 may contribute to reducing the dose and/or frequency of chemo‐ and/or radiotherapy, resulting in an efficient mitigation of the therapy‐related adverse effects. Although normal cells drive the stress response only in the presence of stress, stress response cascades are kept constitutively active in tumor cells in a stress‐independent manner. It is thus anticipated that GO‐Y030‐mediated inhibition of adaptive response could make a stronger impact upon tumor cells than normal cells, suggesting that tumor cells should preferentially be targeted by GO‐Y030. On the other hand, GO‐Y030 may impair tolerance to various stresses in normal cells to some extent, leading to increase of apoptosis. Further studies are required for controlling such an adverse effect to normal cells.

In conclusion, we have shown that GO‐Y030, a curcumin analogue, can efficiently diminish the CSC population and have identified HSP70‐1A and dnaJ‐B1 proteins as direct targets of the GO‐Y030, which inhibits the chaperone function of these targets by reducing their binding activity with a substrate. Our results suggest that GO‐Y030 could impair stress tolerance of CSCs by disrupting the function of HSP70/HSP40 and should finally contribute to a reduction of the CSC population. The present study is the first report to demonstrate the relevance of GO‐Y030 to HSP activity.

## Conflicts of interest

The authors declare that they have no conflicts of interest.

## Author contributions

MS and YO were involved in conceptualization and methodology and wrote the original draft. YO reviewed and edited the draft. MS, YY, ANM, YK, and YO performed the experiments and the data validation. HS was involved in resources. YO supervised the study.

## Supporting information


**Fig. S1.** Effect of GO‐Y030 on release of extracellular HSP70‐1A (eHSP70‐1A) from PC3 cells. To detect eHSP70‐1A in culture medium, an antibody‐sandwich ELISA was performed. PC3 cells were incubated with or without GO‐Y030 (2 μm). After the indicated time intervals, conditioned media were harvested and tested for the presence of eHSP70‐1A by an ELISA. The data are acquired as ratio to control group at 0 h. Statistical significance was calculated using Student's *t*‐test. Data represent the mean ± SEM of four independent experiments. *P < 0.05 compared to the control group at each corresponding time interval.

**Fig. S2.** Effects of GO‐Y030 on global transcription and translation. (A) GSEA was performed to examine a global difference in mRNA expression profile of the indicated gene sets in PC3 cells. Note that GO‐Y030 does not significantly alter them RNA expression level of the genes involved in transcriptional and/or translational machinery. (B) A puromycin incorporation assay was performed to detect polypeptides in the elongation stage during protein synthesis in the presence or absence of GO‐Y030 in PC3 cells. Note that GO‐Y030 does not affect a global translation activity.

**Table S1.** HSP family genes for which the expression was significantly changed by GO‐Y030 pretreatment in PC3 cells. To assess variations in gene expression patterns in GO‐Y030‐treated PC3 cells, a cDNA microarray experiment was carried out. Treatment with GO‐Y030 down‐regulated the mRNA expression of HSP family genes including *HSPA1A* and *DNAJB1*. Fold change is defined as the ratio of expression levels in GO‐Y030‐treated cells versus controls.

## Data Availability

The data that support the findings of this study are available from the corresponding author upon reasonable request.

## References

[1] Jordan CT , Guzman ML , Noble M . Cancer stem cells. N Engl J Med. 2006;355:1253–61. 16990388 10.1056/NEJMra061808

[2] Clarke MF , Dick JE , Dirks PB , Eaves CJ , Jamieson CHM , Jones DL , et al. Cancer stem cells—perspectives on current status and future directions: AACR workshop on cancer stem cells. Cancer Res. 2006;66:9339–44. 16990346 10.1158/0008-5472.CAN-06-3126

[3] Medema JP . Cancer stem cells: the challenges ahead. Nat Cell Biol. 2013;15:338–44. 23548926 10.1038/ncb2717

[4] Singh S , Aggarwal BB . Activation of transcription factor NF‐κB is suppressed by curcumin (diferulolylmethane). J Biol Chem. 1995;270:24995–5000. 7559628 10.1074/jbc.270.42.24995

[5] Huang TS , Lee SC , Lin JK . Suppression of c‐Jun/AP‐1 activation by an inhibitor of tumor promotion in mouse fibroblast cells. Proc Natl Acad Sci USA. 1991;88:5292–6. 1905019 10.1073/pnas.88.12.5292 PMC51858

[6] Chi HP , Eun RH , Park S , Kim HK , Chul HY . The inhibitory mechanism of cur.cumin and its derivative against β‐catenin/Tcf signaling. FEBS Lett. 2005;579:2965–71. 15893313 10.1016/j.febslet.2005.04.013

[7] Mukherjee S , Mazumdar M , Chakraborty S , Manna A , Saha S , Khan P , et al. Curcumin inhibits breast cancer stem cell migration by amplifying the E‐cadherin/β‐catenin negative feedback loop. Stem Cell Res Ther. 2014;5:1–19. 10.1186/scrt506 PMC4445824 25315241

[8] Bharti AC , Donato N , Aggarwal BB . Curcumin (diferuloylmethane) inhibits constitutive and IL‐6‐inducible STAT3 phosphorylation in human multiple myeloma cells. J Immunol. 2003;171:3863–71. 14500688 10.4049/jimmunol.171.7.3863

[9] Chung SS , Vadgama JV . Curcumin and epigallocatechin gallate inhibit the cancer stem cell phenotype via down‐regulation of STAT3‐NFkappaB signaling. Anticancer Res. 2015;35:39–46. 25550533 PMC4290892

[10] Li Y , Zhang T . Targeting cancer stem cells by curcumin and clinical applications. Cancer Lett. 2014;346:197–205. 24463298 10.1016/j.canlet.2014.01.012

[11] Sordillo PP , Helson L . Curcumin and cancer stem cells: curcumin has asymmetrical effects on cancer and normal stem cells. Anticancer Res. 2015;35:599–614. 25667437

[12] Zendehdel E , Abdollahi E , Momtazi‐Borojeni AA , Korani M , Alavizadeh SH , Sahebkar A . The molecular mechanisms of curcumin's inhibitory effects on cancer stem cells. J Cell Biochem. 2019;120:4739–47. 30269360 10.1002/jcb.27757

[13] Sharma RA , Gescher AJ , Steward WP . Curcumin: the story so far. Eur J Cancer. 2005;41:1955–68. 16081279 10.1016/j.ejca.2005.05.009

[14] Ohori H , Yamakoshi H , Tomizawa M , Shibuya M , Kakudo Y , Takahashi A , et al. Synthesis and biological analysis of new curcumin analogues bearing an enhanced potential for the medicinal treatment of cancer. Mol Cancer Ther. 2006;5:2563–71. 17041101 10.1158/1535-7163.MCT-06-0174

[15] Lin L , Liu Y , Li H , Li P‐K , Fuchs J , Shibata H , et al. Targeting colon cancer stem cells using a new curcumin analogue, GO‐Y030. Br J Cancer. 2011;105:212–20. 21694723 10.1038/bjc.2011.200 PMC3142799

[16] Fan G‐C . Role of heat shock proteins in stem cell behavior. Prog Mol Biol Transl Sci. 2012;111:305–22. 22917237 10.1016/B978-0-12-398459-3.00014-9 PMC4422174

[17] Lettini G , Lepore S , Crispo F , Sisinni L , Esposito F , Landriscina M . Heat shock proteins in cancer stem cell maintenance: a potential therapeutic target? Histol Histopathol. 2020;35:25–37. 31322279 10.14670/HH-18-153

[18] Kabakov A , Yakimova A , Matchuk O . Molecular chaperones in cancer stem cells: determinants of stemness and potential targets for antitumor therapy. Cell. 2020;9:892. 10.3390/cells9040892 PMC7226806 32268506

[19] Lamb R , Harrison H , Smith DL , Townsend PA , Jackson T , Ozsvari B , et al. Targeting tumor‐initiating cells: eliminating anabolic cancer stem cells with inhibitors of protein synthesis or by mimicking caloric restriction. Oncotarget. 2015;6:4585–601. 25671304 10.18632/oncotarget.3278 PMC4467101

[20] Lu KT , Wang BY , Chi WY , Chang‐Chien J , Yang JJ , Te LH , et al. Ovatodiolide inhibits breast cancer stem/progenitor cells through SMURF2‐mediated downregulation of Hsp27. Toxins. 2016;8:127. 27136586 10.3390/toxins8050127 PMC4885042

[21] Nishizawa S , Hirohashi Y , Torigoe T , Takahashi A , Tamura Y , Mori T , et al. HSP DNAJB8 controls tumor‐initiating ability in renal cancer stem‐like cells. Cancer Res. 2012;72:2844–54. 22552285 10.1158/0008-5472.CAN-11-3062

[22] Gong J , Weng D , Eguchi T , Murshid A , Sherman MY , Song B , et al. Targeting the hsp70 gene delays mammary tumor initiation and inhibits tumor cell metastasis. Oncogene. 2015;34:5460–71. 25659585 10.1038/onc.2015.1 PMC7331470

[23] Kawasaki Y , Omori Y , Li Q , Nishikawa Y , Yoshioka T , Yoshida M , et al. Cytoplasmic accumulation of connexin32 expands cancer stem cell population in human HuH7 hepatoma cells by enhancing its self‐renewal. Int J Cancer. 2011;128:51–62. 20209499 10.1002/ijc.25308

[24] Iikawa N , Yamamoto Y , Kawasaki Y , Nishijima‐Matsunobu A , Suzuki M , Yamada T , et al. Intrinsic oncogenic function of intracellular Connexin26 protein in head and neck squamous cell carcinoma cells. Int J Mol Sci. 2018;19:2134. 30041406 10.3390/ijms19072134 PMC6073371

[25] Ohi N , Nishikawa Y , Tokairin T , Yamamoto Y , Doi Y , Omori Y , et al. Maintenance of bad phosphorylation prevents apoptosis of rat hepatic sinusoidal endothelial cells in vitro and in vivo. Am J Pathol. 2006;168:1097–106. 16565486 10.2353/ajpath.2006.050462 PMC1606557

[26] Lazarev VF , Sverchinsky DV , Mikhaylova ER , Semenyuk PI , Komarova EY , Niskanen SA , et al. Sensitizing tumor cells to conventional drugs: HSP70 chaperone inhibitors, their selection and application in cancer models. Cell Death Dis. 2018;9:41. 29348557 10.1038/s41419-017-0160-y PMC5833849

[27] Lazarev VF , Onokhin KV , Antimonova OI , Polonik SG , Guzhova IV , Margulis BA . Kinetics of chaperone activity of proteins Hsp70 and Hdj1 in human leukemia U‐937 cells after preconditioning with thermal shock or compound U‐133. Biochemistry. 2011;76:590–5. 21639839 10.1134/S0006297911050099

[28] Castellino FJ , Hill RL . The carboxymethylation of bovine alpha‐lactalbumin. J Biol Chem. 1970;245:417–24. 5460891

[29] Wawrzynów A , Zylicz M . Divergent effects of ATP on the binding of the DnaK and DnaJ chaperones to each other, or to their various native and denatured protein substrates. J Biol Chem. 1995;270:19300–6. 7642605 10.1074/jbc.270.33.19300

[30] Lee CH , Hong HM , Chang YY , Chang WW . Inhibition of heat shock protein (Hsp) 27 potentiates the suppressive effect of Hsp90 inhibitors in targeting breast cancer stem‐like cells. Biochimie. 2012;94:1382–9. 22445681 10.1016/j.biochi.2012.02.034

[31] Chiu CC , Lee LY , Li YC , Chen YJ , Lu YC , Li YL , et al. Grp78 as a therapeutic target for refractory head‐neck cancer with CD24^−^ CD44^+^ stemness phenotype. Cancer Gene Ther. 2013;20:606–15. 24201869 10.1038/cgt.2013.64

[32] Hsu HS , Lin JH , Huang WC , Hsu TW , Su K , Chiou SH , et al. Chemoresistance of lung cancer stemlike cells depends on activation of Hsp27. Cancer. 2011;117:1516–28. 21425153 10.1002/cncr.25599

[33] Newman B , Liu Y , Lee HF , Sun D , Wang Y . HSP90 inhibitor 17‐AAG selectively eradicates lymphoma stem cells. Cancer Res. 2012;72:4551–61. 22751135 10.1158/0008-5472.CAN-11-3600 PMC3443561

[34] Subramanian C , Kovatch KJ , Sim MW , Wang G , Prince ME , Carey TE , et al. Novel C‐terminal heat shock protein 90 inhibitors (KU711 and Ku757) are effective in targeting head and neck squamous cell carcinoma cancer stem cells. Neoplasia. 2017;19:1003–11. 29121598 10.1016/j.neo.2017.09.003 PMC5681325

[35] Le HT , Nguyen HT , Min HY , Hyun SY , Kwon S , Lee Y , et al. Panaxynol, a natural Hsp90 inhibitor, effectively targets both lung cancer stem and non‐stem cells. Cancer Lett. 2018;412:297–307. 29061506 10.1016/j.canlet.2017.10.013

[36] Leu JIJ , Pimkina J , Frank A , Murphy ME , George DL . A small molecule inhibitor of inducible heat shock protein 70. Mol Cell. 2009;36:15–27. 19818706 10.1016/j.molcel.2009.09.023 PMC2771108

[37] Williamson DS , Borgognoni J , Clay A , Daniels Z , Dokurno P , Drysdale MJ , et al. Novel adenosine‐derived inhibitors of 70 kDa heat shock protein, discovered through structure‐based design. J Med Chem. 2009;52:1510–3. 19256508 10.1021/jm801627a

[38] Massey AJ , Williamson DS , Browne H , Murray JB , Dokurno P , Shaw T , et al. A novel, small molecule inhibitor of Hsc70/Hsp70 potentiates Hsp90 inhibitor induced apoptosis in HCT116 colon carcinoma cells. Cancer Chemother Pharmacol. 2010;66:535–45. 20012863 10.1007/s00280-009-1194-3

[39] Li X , Srinivasan SR , Connarn J , Ahmad A , Young ZT , Kabza AM , et al. Analogues of the allosteric heat shock protein 70 (Hsp70) inhibitor, MKT‐077, As anti‐cancer agents. ACS Med Chem Lett. 2013;4:1042–7. 24312699 10.1021/ml400204n PMC3845967

[40] Thorsteinsdottir J , Stangl S , Fu P , Guo K , Albrecht V , Eigenbrod S , et al. Overexpression of cytosolic, plasma membrane bound and extracellular heat shock protein 70 (Hsp70) in primary glioblastomas. J Neurooncol. 2017;135:443–52. 28849427 10.1007/s11060-017-2600-z

[41] Wu FH , Yuan Y , Li D , Liao SJ , Yan B , Wei JJ , et al. Extracellular HSPA1A promotes the growth of hepatocarcinoma by augmenting tumor cell proliferation and apoptosis‐resistance. Cancer Lett. 2012;317:157–64. 22115967 10.1016/j.canlet.2011.11.020

[42] Komarova EY , Marchenko LV , Zhakhov AV , Nikotina AD , Aksenov ND , Suezov RV , et al. Extracellular hsp70 reduces the pro‐tumor capacity of monocytes/macrophages co‐cultivated with cancer cells. Int J Mol Sci. 2020;21:59. 10.3390/ijms21010059 PMC6982218 31861801

[43] Fan CY , Lee S , Cyr DM . Mechanisms for regulation of Hsp70 function by Hsp40. Cell Stress Chaperones. 2003;8:309–16. 15115283 10.1379/1466-1268(2003)008<0309:mfrohf>2.0.co;2 PMC514902

[44] Kampinga HH , Craig EA . The HSP70 chaperone machinery: J proteins as drivers of functional specificity. Nat Rev Mol Cell Biol. 2010;11:579–92. 20651708 10.1038/nrm2941 PMC3003299

[45] Palleros DR , Welch WJ , Fink AL . Interaction of hsp70 with unfolded proteins: effects of temperature and nucleotides on the kinetics of binding. Proc Natl Acad Sci USA. 1991;88:5719–23. 1829527 10.1073/pnas.88.13.5719 PMC51949

[46] Langer T , Lu C , Echols H , Flanagan J , Hayer MK , Hartl FU . Successive action of DnaK, DnaJ and GroEL along the pathway of chaperone‐mediated protein folding. Nature. 1992;356:683–9. 1349157 10.1038/356683a0

[47] Hyun SY , Le HT , Min HY , Pei H , Lim Y , Song I , et al. Evodiamine inhibits both stem cell and non‐stem‐cell populations in human cancer cells by targeting heat shock protein 70. Theranostics. 2021;11:2932–52. 33456581 10.7150/thno.49876 PMC7806467

