# Supplementary material for: A curcumin analogue GO‐Y030 depletes cancer stem cells by inhibiting the interaction between the HSP70/HSP40 complex and its substrates
Source: FEBS Open Bio. 2023 Jan 24;13(3):434–46. doi: 10.1002/2211-5463.13550 (PMC9989923; doi:10.1002/2211-5463.13550)
Supplement: Supplementary file 1 — Fig. S1. Effect of GO‐Y030 on release of extracellular HSP70‐1A (eHSP70‐1A) from PC3 cells. To detect eHSP70‐1A in culture medium, an antibody‐sandwich ELISA was performed. PC3 cells were incubated with or without GO‐Y030 (2 μm). After the indicated time intervals, conditioned media were harvested and tested for the presence of eHSP70‐1A by an ELISA. The data are acquired as ratio to control group at 0 h. Statistical significance was calculated using Student's t‐test. Data represent the mean ± SEM of four independent experiments. *P < 0.05 compared to the control group at each corresponding time interval. [file FEB4-13-434-s002.pdf]

Suzuki et al.
Figure S1

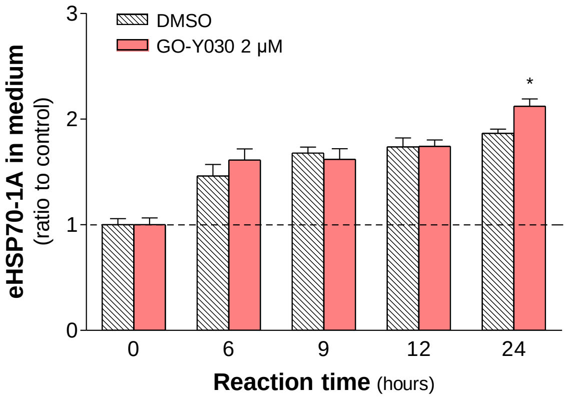

Fig. S1. Effect of GO-Y030 on release of extracellular HSP70-1A (eHSP70-1A) from PC3 cells. To detect eHSP70-1A in culture medium, an antibody-sandwich ELISA was performed. PC3 cells were incubated with or without GO-Y030 (2 μM). After indicated time intervals, conditioned media were harvested and tested for the presence of eHSP70-1A by ELISA. The data are acquired as ratio to control group at 0 hour. Statistical significance was calculated using Student's t-test. The data represented as means ± SEM of four independent experiments. *$P < 0.05$ compared with control group at each corresponding time interval.
