# Supplementary material for: A curcumin analogue GO‐Y030 depletes cancer stem cells by inhibiting the interaction between the HSP70/HSP40 complex and its substrates
Source: FEBS Open Bio. 2023 Jan 24;13(3):434–46. doi: 10.1002/2211-5463.13550 (PMC9989923; doi:10.1002/2211-5463.13550)
Supplement: Supplementary file 2 — Fig. S2. Effects of GO‐Y030 on global transcription and translation. (A) GSEA was performed to examine a global difference in mRNA expression profile of the indicated gene sets in PC3 cells. Note that GO‐Y030 does not significantly alter them RNA expression level of the genes involved in transcriptional and/or translational machinery. (B) A puromycin incorporation assay was performed to detect polypeptides in the elongation stage during protein synthesis in the presence or absence of GO‐Y030 in PC3 cells. Note that GO‐Y030 does not affect a global translation activity. [file FEB4-13-434-s003.pdf]

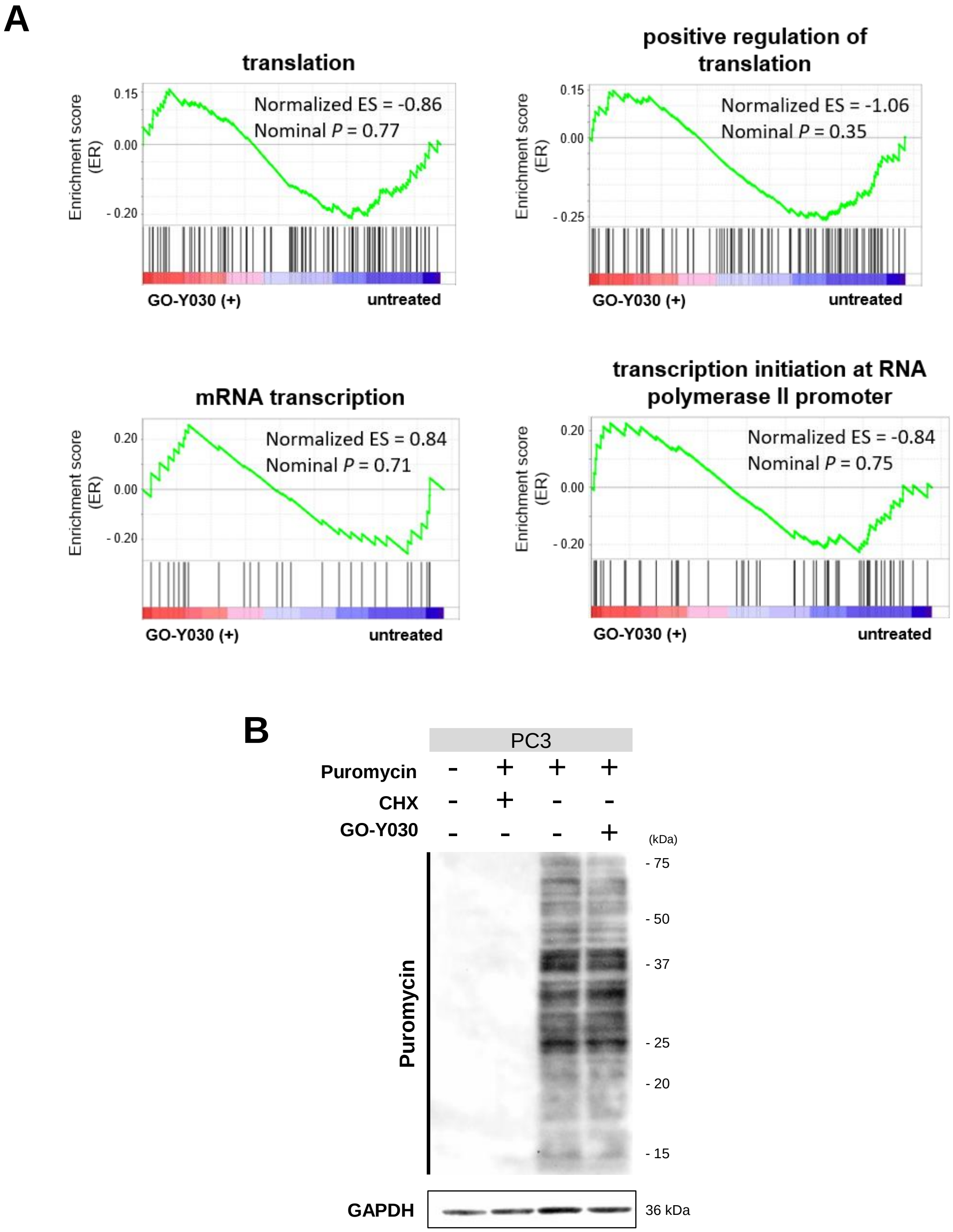

Fig. S2. Effects of GO-Y030 on global transcription and translation. **(A)** GSEA was performed to examine a global difference in mRNA expression profile of the indicated gene sets in PC3 cells. Note that GO-Y030 does not significantly alter the mRNA expression level of the genes involved in transcriptional and/or translational machinery. **(B)** Puromycin incorporation assay was performed to detect polypeptides in the elongation stage during protein synthesis in the presence or absence of GO-Y030 in PC3 cells. Note that GO-Y030 does not affect a global translation activity.
