# Supplementary material for: A curcumin analogue GO‐Y030 depletes cancer stem cells by inhibiting the interaction between the HSP70/HSP40 complex and its substrates
Source: FEBS Open Bio. 2023 Jan 24;13(3):434–46. doi: 10.1002/2211-5463.13550 (PMC9989923; doi:10.1002/2211-5463.13550)
Supplement: Supplementary file 3 — Table S1. HSP family genes for which the expression was significantly changed by GO‐Y030 pretreatment in PC3 cells. To assess variations in gene expression patterns in GO‐Y030‐treated PC3 cells, a cDNA microarray experiment was carried out. Treatment with GO‐Y030 down‐regulated the mRNA expression of HSP family genes including HSPA1A and DNAJB1. Fold change is defined as the ratio of expression levels in GO‐Y030‐treated cells versus controls. [file FEB4-13-434-s001.docx]

| **Accession no.** | **symbol** | **description** | **Fold**  **change** | **logFC** |
| --- | --- | --- | --- | --- |
| NM_005345.5 | HSPA1A | heat shock protein family A (Hsp70) member 1A | 0.4 | -1.32 |
| NM_001300914.1 | DNAJB1 | DnaJ heat shock protein family (Hsp40) member B1 | 0.57 | -0.81 |
| NM_001539.3 | DNAJA1 | DnaJ heat shock protein family (Hsp40) member A1 | 0.6 | -0.74 |
| XM_011542798.1 | HSPA8 | heat shock protein family A (Hsp70) member 8 | 0.61 | -0.72 |
| XM_011536718.1 | HSP90AA1 | heat shock protein 90kDa alpha family class A member 1 | 0.71 | -0.49 |

**Table S1.** HSP family genes the expression of which was significantly changed by GO-Y030 pretreatment in PC3 cells.

To assess variations in gene expression patterns in GO-Y030-treated PC3 cells, cDNA microarray experiment was carried out. Treatment with GO-Y030 down-regulated the mRNA expression of HSP family genes including *HSPA1A* and *DNAJB1*. Fold change is defined as the ratio of expression levels in GO-Y030-treated cells versus controls.
